# Negative Regulation of C/EBPbeta1 by Sumoylation in Breast Cancer Cells

**DOI:** 10.1371/journal.pone.0025205

**Published:** 2011-09-28

**Authors:** Allison A. Atwood, Rachel Jerrell, Linda Sealy

**Affiliations:** 1 Department of Cancer Biology, Vanderbilt University School of Medicine, Nashville, Tennessee, United States of America; 2 Department of Molecular Physiology and Biophysics, Vanderbilt University School of Medicine, Nashville, Tennessee, United States of America; National Taiwan University Hosipital, Taiwan

## Abstract

Sumoylation is a post-translational modification that is oftentimes deregulated in diseases such as cancer. Transcription factors are frequent targets of sumoylation and modification by SUMO can affect subcellular localization, transcriptional activity, and stability of the target protein. C/EBPbeta1 is one such transcription factor that is modified by SUMO-2/3. Non-sumoylated C/EBPbeta1, p52-C/EBPbeta1, is expressed in normal mammary epithelial cells but not breast cancer cell lines and plays a role in oncogene-induced senescence, a tumor suppressive mechanism. Although p52-C/EBPbeta1 is not observed via immunoblot in breast cancer cell lines, higher molecular weight bands are observed when breast cancer cell lines are subjected to immunoblot analysis with a C/EBPbeta1-specific antibody. We show that exogenously expressed C/EBPbeta1 is sumoylated in breast cancer cells, and that the higher molecular weight bands we observe in anti-C/EBPbeta1 immunoblots of breast cancer cell lines is sumoylated C/EBPbeta1. Phosphorylation oftentimes enhances sumoylation, and phosphorylation cascades are activated in breast cancer cells. We demonstrate that phosphorylation of C/EBPbeta1Thr235 by Erk-2 enhances sumoylation of C/EBPbeta1 *in vitro*. In addition, sumoylated C/EBPbeta1 is phosphorylated on Thr235 and mutation of Thr235 to alanine leads to a decrease in sumoylation of C/EBPbeta1. Finally, using a C/EBPbeta1-SUMO fusion protein we show that constitutive sumoylation of C/EBPbeta1 completely blocks its capability to induce senescence in WI38 fibroblasts expressing hTERT. Thus, sumolylation of C/EBPbeta1 in breast cancer cells may be a mechanism to circumvent oncogene-induced senescence.

## Introduction

The post-translational modification sumoylation regulates the function of a growing list of proteins that have roles in a variety of cell processes. Because of this, deregulation of the SUMO pathway has been observed in numerous diseases including neurodegenerative disorders [1] diabetes [2], and cancer [3]. Four members of the SUMO (Small Ubiquitin-like MOdifier) family have been identified, SUMO-1, -2, -3, and -4, all of which share homology with ubiquitin [4]. SUMO-2 and SUMO-3 only differ from one another by three amino acid residues and are viewed as being functionally identical. SUMO-2/3 are 50% identical to SUMO-1 [5]. Much less is known about SUMO-4 than the first three members of the SUMO family. SUMO proteins are a group of polypeptides that conjugate to the lysine residue within the target four amino acid consensus sequence: large, hydrophobic amino acid, lysine, alanine, glutamate. SUMO is conjugated to target proteins in much the same manner as ubiquitin. A SUMO-activating enzyme (E1) carries out an ATP-dependent activation of the SUMO carboxy terminus and then transfers the activated SUMO to the SUMO-conjugating enzyme (E2), also known as Ubc9. SUMO is then transferred from Ubc9 to the target, oftentimes with the assistance of one of several SUMO E3 ligases [4], [5]. The SUMO peptides are about 11 kDa but they appear larger on SDS-PAGE gels and can add as much as 20 kDa to the apparent molecular weight of substrates [5]. SUMO-2/3 are able to form chains on target proteins because the SUMO-2/3 peptide contains a SUMO consensus site. It is thought that most of the SUMO-1 in cells is conjugated to proteins whereas free pools exist of the more abundant SUMO-2/3 [6]. SUMO-2/3 is believed to be utilized when cells are exposed to a variety of stresses. The bulk of SUMO substrates that have been identified are involved in chromatin organization, transcription, RNA metabolism, and cytoplasm-nuclear transport [6]. Sumoylation has been shown to be involved in maintenance of genome integrity, protein localization, inhibiting ubiquitination, and regulation of transcription, among other cellular functions [5].

CCAAT/enhancer binding protein beta (C/EBPbeta) contains a SUMO consensus site within its sequence centered around lysine 173 [7]. C/EBPbeta is a basic leucine zipper transcription factor in which three protein isoforms exist due to alternative translation initiation at three in-frame methionines. In humans, full-length C/EBPbeta1 begins at the first in-frame methionine, is 346 amino acids long (297 in rat and mouse) and has an apparent molecular weight of 52 kDa. C/EBPbeta2 begins at the second in-frame ATG, 23 amino acids (21 in rat and mouse) downstream from the first, and appears as a doublet on immunoblots at 45 kDa and 48 kDa. C/EBPbeta3 starts at the final in-frame methionine at amino acid 198 in humans and has an apparent molecular weight of 20 kDa. C/EBPbeta1 and C/EBPbeta2 both contain the C-terminal DNA binding/dimerization domain as well as an N-terminal transactivation domain, allowing them to function as activators of transcription. C/EBPbeta3 is missing the N-terminal transactivation domain and is thus a repressor of transcription [8]. It has been proposed that these three isoforms arise through the presence of alternative translation initiation sites via a leaky ribosome scanning mechanism [8], [9]. A small upstream open reading frame has also been demonstrated to be of importance in regulating translation of C/EBPbeta2 [10]. There is further evidence that C/EBPbeta3 can be produced via proteolytic degradation of the longer isoforms [11].

C/EBPbeta is expressed and plays important roles in a wide number of tissue types. The C/EBPbeta knockout mouse demonstrated that C/EBPbeta plays an essential role in the development of the mammary gland. These mice display a defect in mammary epithelial cell proliferation in response to hormonal stimulation at puberty or pregnancy and a defect in mammary epithelial cell differentiation in response to lactation specific hormones [12], [13]. The production of multiple isoforms of C/EBPbeta may explain how a single transcription factor can regulate various functions. There is accumulating evidence that the three isoforms of C/EBPbeta are functionally distinct. C/EBPbeta3 has recently been found to play a role in autophagy and cell death in breast cancer cells [14]. On the contrary, C/EBPbeta2 has been shown to promote cell growth, transformation, and invasiveness of the immortalized MCF10A mammary epithelial cell line [15], [16]. C/EBPbeta2 is not expressed in normal mammary epithelial tissue from reduction mammoplasty, however high expression of this second isoform is observed in primary human breast cancer tissue [17], [18]. Additionally, C/EBPbeta2 is expressed in breast cancer cell lines and can transactivate cyclin D1 and PLAC1, two genes whose protein products are involved in proliferation and commonly upregulated in breast cancer [17], [18]. In stark contrast to C/EBPbeta2, the first isoform of C/EBPbeta is expressed in normal mammary epithelial tissue from reduction mammoplasty but not in breast cancer cell lines [17], [18]. The C/EBPbeta1 isoform has been implicated in the differentiation of myeloid cells through activation of genes involved in differentiation such as mim-1. This activation was attributed to the ability of C/EBPbeta1 to interact with and recruit the SWI/SNF chromatin remodeling complex [19]. Moreover, C/EBPbeta has been shown to be an essential player in onogene-induced senescence, a tumor suppressive mechanism [20], [21]. A recent report indicates that C/EBPbeta1 is the isoform responsible for the induction senescence [22], [23].

Interestingly, C/EBPbeta1 is the only transactivator isoform of C/EBPbeta that is sumoylated by SUMO-2/3 in Cos-7 cells even though both C/EBPbeta1 and C/EBPbeta2 contain the SUMO consensus sequence around lysine 173 [7]. It has been demonstrated that C/EBPbeta-1 can be sumoylated on lysine 173 and the first 23 amino acids unique to C/EBPbeta-1 are necessary for efficient sumoylation. Mutation of this target lysine 173 to an alanine did not affect sub-nuclear localization of C/EBPbeta-1 [7]. Sumoylation of transcription factors frequently causes transcriptional repression. This transcriptional repression is oftentimes due to sumoylation leading to an alteration in binding partners. The sumoylated protein interacts with transcriptional co-repressors such as histone deacetylases (HDACs), Daxx, members of the NURD co-repressor complex, and Polycomb group proteins [24]. Elk-1 [25], PPAR-gamma [26] and Pax3 [27] are examples where sumoylation of these transcription factors led to their association with transcriptional co-repressors and consequently repression of target genes. Although expression of the 52 kDa form of C/EBPbeta1 is not observed in breast cancer cells, sumoylated C/EBPbeta1 would migrate more slowly via SDS-PAGE resulting in higher molecular weight bands. Since sumoylation oftentimes leads to transcriptional repression of target proteins, negative regulation of C/EBPbeta1 by sumoylation would give cancer cells a growth advantage since C/EBPbeta1 plays a role in oncogene-induced senescence, a tumor suppressive mechanism.

Additionally, phosphorylation of target proteins oftentimes enhances sumoylation. Examples of this include phosphorylation and subsequent enhancement of sumoylation of STAT1 [28], PPAR-gamma [29], MEF2 [30], [31], and Estrogen-related receptor alpha-1 [32]. Phosphorylation cascades known to phosphorylate C/EBPbeta are activated in breast cancer cells. For example, the Ras pathway is activated in most breast cancer cells via activation of upstream receptors, activation of Ras itself, or activation of downstream kinases [33]. Activation of the Ras pathway leads to the activation of numerous kinases that phosphorylate C/EBPbeta on Threonine 235 (Thr235) including Erk-2 [34], cdk2 [35], [36], and p38 [37], [38]. Therefore, phosphorylation of C/EBPbeta1 on Thr235 in transformed cells may enhance sumoylation, thus repressing the transcriptional ability of C/EBPbeta1 to induce senescence. In the current study we demonstrate that an antibody specific to C/EBPbeta1 recognizes higher molecular weight bands in a panel of breast cancer cell lines. When C/EBPbeta1 is exogenously expressed in breast cancer cells, sumoylation of C/EBPbeta1 is evident. Importantly we show that the higher molecular weight bands in breast cancer cell lines recognized by the C/EBPbeta1-specific antibody are sumoylated C/EBPbeta1. Additionally, phosphorylation of purified C/EBPbeta1 by Erk-2 enhances sumoylation, in vitro, and sumoylated C/EBPbeta1 is phosphorylated on Thr235. Furthermore, mutation of C/EBPbeta1Thr235 to alanine, thus preventing phosphorylation of this residue, leads to a decrease in sumoylation of C/EBPbeta1. Finally, a C/EBPbeta1-SUMO fusion protein is completely incapable of inducing senescence in WI38 fibroblasts whereas C/EBPbeta1 effectively induces senescence. Taken together, our results indicate that activated Ras signaling in breast cancer cells may lead to the sumoylation of C/EBPbeta1 and concomitant inactivation of its ability to induce senescence, thereby comprising a means to escape OIS.

## Results

### Sumoylation of C/EBPbeta1 in breast cancer cell lines

**Figure 1:**
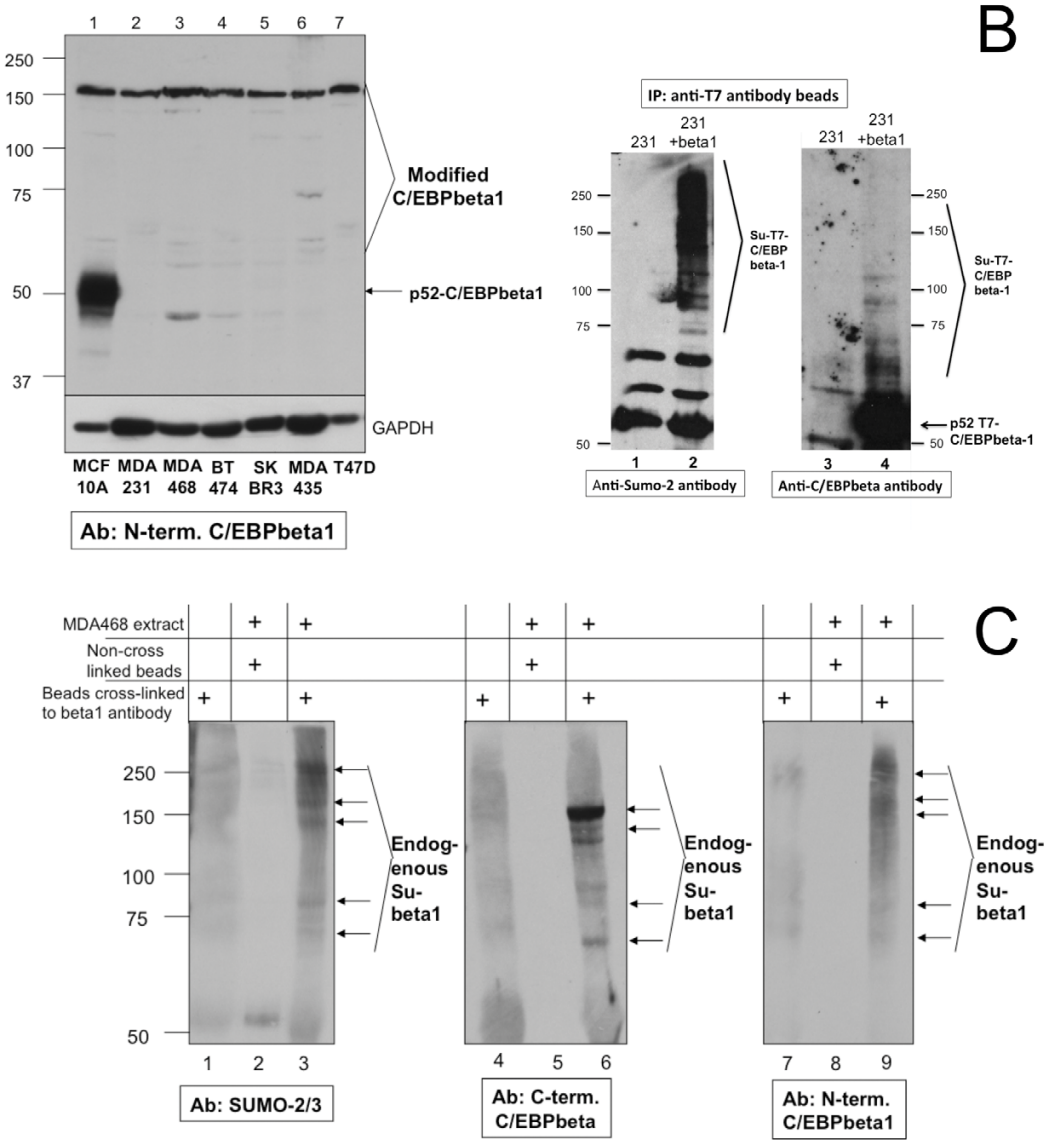
Sumoylation of C/EBPbeta1 in breast cancer cells. a. Cell lysates were prepared and were run on an 8% SDS-PAGE in the following order: lane 1 MCF10A, lane 2 MDA231, lane 3 MDA468, lane 4 BT-474, lane 5 SK-BR3, lane 6 MDA435 and lane 7 T47D. Immunoblot analysis was performed with a C/EBPbeta1-specific antibody raised to the first 21 amino acids unique to C/EBPbeta1 (Abcam 18F8). The bottom immunoblot was performed as a loading control for GAPDH. Bars indicate the mobility's of standard molecular weight markers, in kilo-Daltons (kDa), in all figures. b. MDA231 breast cancer cells were infected with T7-C/EBPbeta1-IRES-eGFP-LZRS three times and sorted by FACs using GFP as a marker. Immunoprecipitations of confluent 100 mm dishes were performed with uninfected MDA231s (lanes 1 and 3) or T7-C/EBPbeta1-MDA231 cells (lanes 2 and 4) using T7 antibody beads. The left is an immunoblot with an anti-SUMO-2/3 antibody and the immunoblot on the right is with an anti-C/EBPbeta antibody. Sumoylated C/EBPbeta1 is indicated and the parent p52 C/EBPbeta1 is indicated by the arrow. c. Immunoprecipitations of MDA468 cells were performed with protein A agarose beads cross-linked to a C/EBPbeta1-specific antibody (described in [7]). Lanes 3, 6, and 9 are the immunoprecipitations whereas lanes 1, 4, and 7 are negative control beads only and lanes 2, 5 and 8 are negative control non-crosslinked beads incubated with MDA468 extract. The left immunoblot is performed with an anti-SUMO-2/3 antibody, the middle immunoblot with a C-terminal C/EBPbeta antibody (Abcam 47A1) and the right hand immunoblot with a C/EBPbeta1-specifc antibody (Abcam 18F8). Arrows indicate sumoylated C/EBPbeta1. (231 = MDA231, beta1 = C/EBPbeta1, su = sumoylated).

p52-C/EBPbeta1 is not observed via immunoblot analysis of breast cancer cell lines [17], Figure 1a, however p52-C/EBPbeta1 is expressed in the MCF10A immortalized but non-transformed mammary epithelial cell line [Figure 1a]. Figure 1a utilizes an antibody raised to the first 23 amino acids present only in the first isoform of C/EBPbeta. Using this C/EBPbeta1-specific antibody, immunoblot analysis of a panel of breast cancer cells results in higher molecular weight bands. These higher molecular weight bands are likely post-translationally modified C/EBPbeta1, because C/EBPbeta1 can be modified by a variety of post-translational modifications known to increase the apparent molecular weight of the protein via SDS-PAGE. It is likely that these bands are not non-specific, as antibodies raised to other regions of C/EBPbeta also recognize these higher molecular weight bands (Figure 1c and data not shown).

Knowing that C/EBPbeta1 can be sumoylated in Cos-7 cells and after observing higher molecular weight bands on the immunoblots of breast cancer cells using the C/EBPbeta1-specific antibody, we wanted to know if C/EBPbeta1 could be sumoylated in breast cancer cell lines. We expressed T7-tagged C/EBPbeta1 (T7-C/EBPbeta1) by infecting breast cancer cells with a retroviral vector expressing T7-C/EBPbeta1-IRES-eGFP. Infected cells were then sorted by fluoresecence activated cell sorting (FACS) using green fluorescent protein (GFP) as a marker resulting in a homogenous population of cells expressing T7-C/EBPbeta1 and GFP. Immunoprecipitations were performed with T7 antibody beads and resulting immunoblot analysis reveals that C/EBPbeta1 is sumoylated when exogenously expressed in the MDA231 breast cancer cell line (Figure 1b). The immunoblot on the left is with an anti-SUMO-2/3 antibody, and there are distinct higher molecular weight bands in the MDA231-T7-C/EBPbeta1 lane as compared to control MDA231 only (lane 2 compared to lane1). These bands coincide with bands in the MDA231-T7-C/EBPbeta1 lane (lane 4) on the immunoblot on the right in which an anti-C/EBPbeta antibody is used. Similar results were obtained using the SKBR3 and HCC1954 breast cancer cell lines (data not shown), allowing us to conclude that sumoylation of C/EBPbeta1 occurs in breast cancer cells.

Next we wanted to determine if the higher molecular weight bands that are observed in the anti-C/EBPbeta1 immunoblot in Figure 1a are sumoylated C/EBPbeta1. To examine this, we used our C/EBPbeta1-specific antibody described in Eaton et al, 2001 cross-linked to protein A beads. Immunoprecipitations were then performed with these beads using cell extracts from MDA468 and MDA231 breast cancer cell lines. The results from the MDA468 immunoprecipitation are shown in Figure 1c. The immunoblot on the left uses an anti-SUMO-2/3 specific antibody, the middle immunoblot is with a C-terminal C/EBPbeta antibody, and the immunoblot on the right is with the C/EBPbeta1-specific antibody. Lanes 3, 6, and 9 are the immunoprecipitations with MDA468 extract, whereas the other lanes are negative controls. Lanes 3, 6, and 9 exhibit higher molecular weight bands that correspond in mobility and are not present in the lanes with control samples. The anti-SUMO-2/3 antibody recognizes these unique higher molecular weight bands (Figure 1c lane 3), which line up with the bands in the anti-C/EBPbeta immunoblots (Figure 1c, lanes 6 and 9), thus demonstrating that endogenous C/EBPbeta1 is sumoylated in breast cancer cell lines.

### C/EBPbeta1 is phosphorylated on Thr235 by Erk2 and this phosphorylation enhances sumoylation of C/EBPbeta1

**Figure 2:**
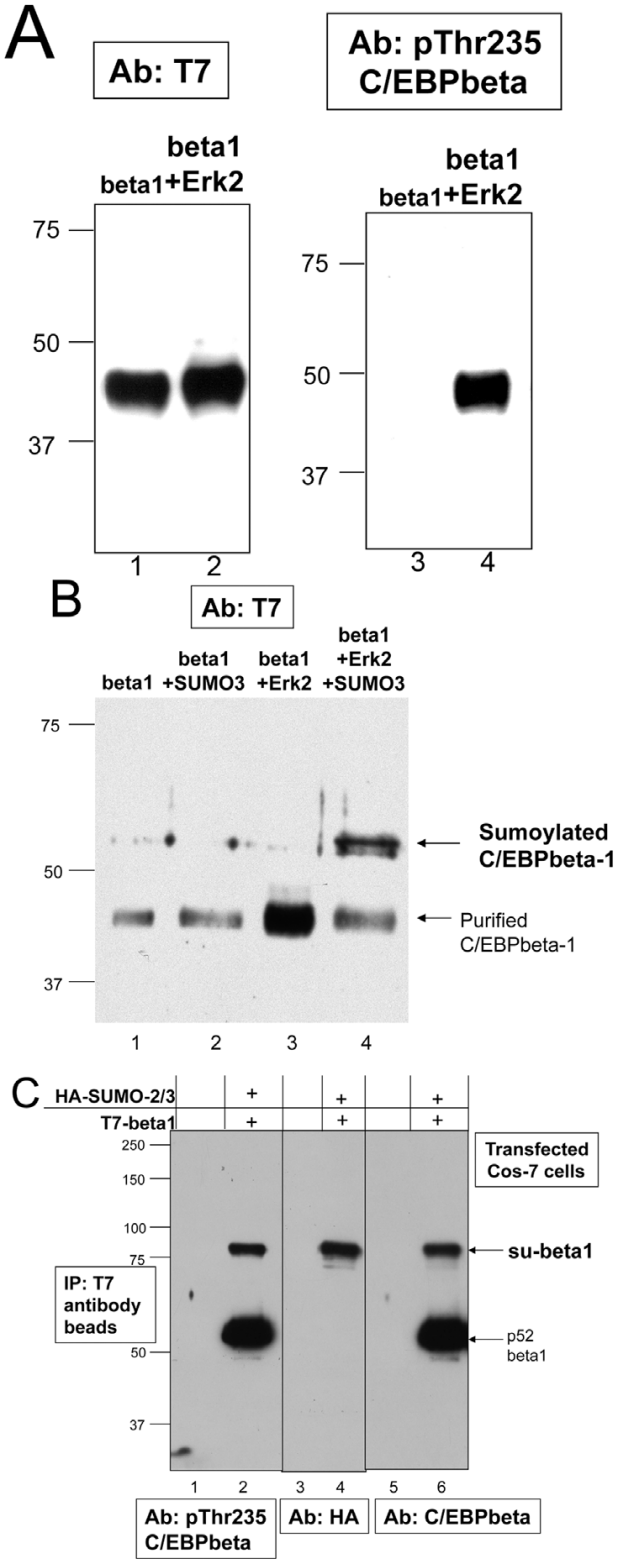
Phosphorylation of C/EBPbeta1 by Erk-2 enhances sumoylation *in vitro* and sumoylated C/EBPbeta1 is phosphorylated on Thr235. a. Immunoblot analysis of purified rat C/EBPbeta1 (Lap1) (lanes 1 and 3) and C/EBPbeta1 incubated with purified, active Erk-2 (lanes 2 and 4). The immunoblot on the left is with the anti-T7 tag antibody and on the right is with the anti-phosphoThr235 C/EBPbeta antibody. Rat C/EBPbeta1 migrates faster via SDS-PAGE because it is smaller in size than human C/EBPbeta1. b. Immunoblot analysis with the anti-T7 tag antibody. Lane 1 is purified rat C/EBPbeta1, lane 2 is C/EBPbeta1 incubated with purified E1 SUMO activating enzyme, purified E2 SUMO conjugating enzyme, and purified SUMO-3 peptide, and lane 3 is C/EBPbeta1 with Erk-2, E1, E2 and SUMO-3. Arrows indicate C/EBPbeta1 and sumoylated C/EBPbeta1. c. Cos-7 cells were untransfected (lanes 1, 3, and 5) or transfected with T7-C/EBPbeta1-pcDNA3.1 and HA-SUMO-2-pcDNA3 (lanes 2, 4 and 6). All samples were immunoprecipiated with T7 antibody beads. Immunoblot analysis was performed with the anti-phosphoThr235 C/EBPbeta antibody (left), anti-HA tag (middle), and anti-C/EBPbeta antibody (right). Arrows indicate sumoylated T7-C/EBPbeta1 and p52-T7-C/EBPbeta1. (beta1 = C/EBPbeta1).

It is well-known that C/EBPbeta is phosphorylated on Thr235 by Erk-2, however few studies have examined which particular isoform of C/EBPbeta is phosphorylated by Erk-2. One study has determined that C/EBPbeta2 is phosphorylated by Erk-2 on Thr235 [34], but no one has determined whether C/EBPbeta1 can be phosphorylated on this residue by Erk-2. To examine this, we took purified rat C/EBPbeta1 protein and incubated it with purified, active Erk-2. Figure 2a, right panel, is an immunoblot with an anti-pThr235 C/EBPbeta-specific antibody illustrating that C/EBPbeta1 is phosphorylated on Thr235 after incubation with Erk-2 (compare lane 4 with lane 3), *in vitro*. Figure 2a, left panel, is the same immunoblot only with the anti-T7 tag antibody demonstrating approximately equal amounts of protein are present in both lanes (compare lanes 1 and 2). Rat C/EBPbeta is 298 amino acids compared to human C/EBPbeta which is 345 amino acids. Consequently, rat C/EPbeta1 (also termed LAP1) migrates with an apparent molecular weight of 45 kD (Figure 2a,b) compared to human C/EBPbeta1 at 52 kD in the human breast cancer cell lines (Figure 1) or CMV-driven expression vector (Figure 2c).

After determining that Erk-2 phosphorylates C/EBPbeta1 on Thr235 *in vitro*, we wanted to determine if this phosphorylation could enhance sumoylation of C/EBPbeta1, as this has been found to be true for several other transcription factors. To do this we incubated purified C/EBPbeta1 protein with Erk-2, followed by the addition of purified SUMO-2/3 peptide, the SUMO activating (E1) enzyme, and the SUMO conjugating (E2) enzyme. Figure 2b is an immunoblot with the anti-T7 tag antibody indicating that incubation with Erk-2 prior to incubation with SUMO-2/3 enhances sumoylation of C/EBPbeta1 (compare lanes 2 and 4). Although sumoylation is enhanced, very high molecular weight bands, 75 kD or greater typical of C/EBPbeta1 sumoylated in vivo, are not observed in this in vitro assay. This is likely because sumoylation in this purified system is inefficient, quite possibly due to the absence of any E3 ligases which are known to stimulate sumoylation in vivo.

Next we wanted to determine if sumoylated C/EBPbeta1 is phosphorylated on Thr235. T7-C/EBPbeta1 and HA-SUMO-2/3 were transiently transfected into Cos-7 cells and immunoprecipitations were performed with T7 antibody beads. The anti-phosphoThr235 C/EBPbeta antibody was used on the immunoblot in Figure 2c, lanes 1 and 2. Figure 2c, lane 2 demonstrates that sumoylated C/EBPbeta1 is phosphorylated on Thr235. The middle immunoblot is with the anti-HA tag antibody (Figure 2c. lanes 3 and 4) confirming that the higher molecular weight band is sumoylated C/EBPbeta1 and the immunoblot on the right is with the anti-C/EBPbeta C-terminal antibody (Figure 2c, lanes 5 and 6).

### Mutation of Thr235 to alanine decreases sumoylation of C/EBPbeta1

**Figure 3:**
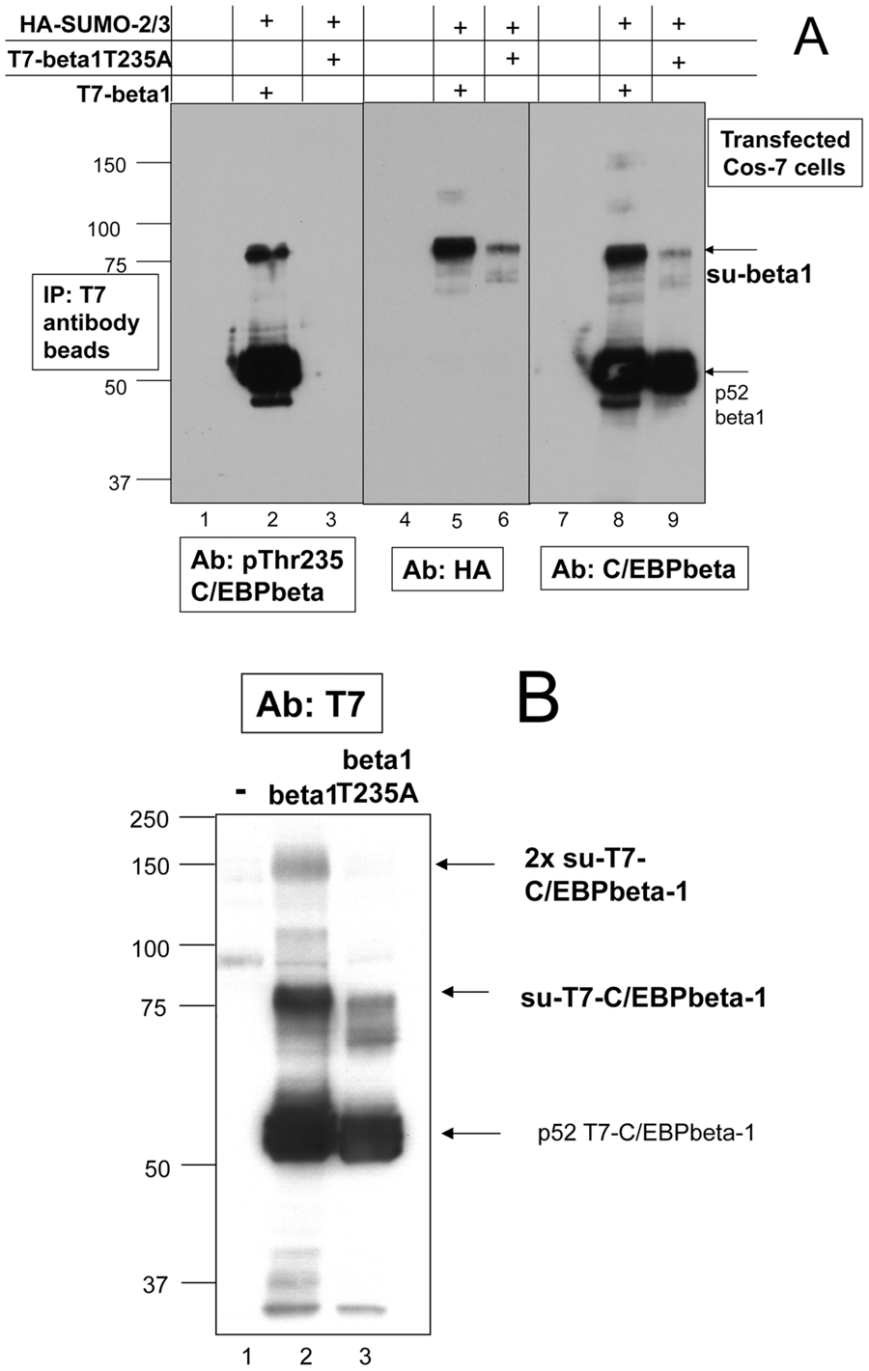
Mutation of Thr235 to alanine decreases sumoylation of C/EBPbeta1. a. Cos-7 cells were untransfected (lanes 1, 4, and 7), transfected with T7-C/EBPbeta1-pcDNA3.1 and HA-SUMO-2-pcDNA3 (lanes 2, 5 and 8), or transfected with T7-C/EBPbeta1T235A-pcDNA3.1 and HA-SUMO-2-pcDNA3 (lanes 3, 6, and 9). All samples were immunoprecipitated with T7 antibody beads. Immunoblot analysis was performed with the anti-phosphoThr235 C/EBPbeta antibody (left), anti-HA tag (middle), and anti-C/EBPbeta antibody (right). Arrows indicate sumoylated T7-C/EBPbeta1 and p52-T7-C/EBPbeta1. b. Immunoblot analysis using the anti-T7 tag antibody of cell lysates from Cos-7 (lane 1), Cos-7 cells transfected with T7-C/EBPbeta1-pcDNA3.1 and HA-SUMO-2-pcDNA3 (lane 2), and Cos-7 transfected with T7-C/EBPbeta1T235A-pcDNA3.1 and HA-SUMO-2-pcDNA3 (lane 3). Arrows indicate p52-T7-C/EBPbeta1 and sumoylated T7-C/EBPbeta1. The relative amount of protein in the parent T7-C/EBPbeta1 band and the 75 kDa sumoylated T7-C/EBPbeta1 band was measured using the LI-COR Odyssey system. It was determined that there is 3.25 times more sumoylated 75 kDa T7-C/EBPbeta1 as there is 75 kDa T7-C/EBPbeta1T235A. This was calculated relative to the p52-T7-C/EBPbeta1 and p52-T7-C/EBPbeta1T235A bands. This was repeated three times with a standard deviation of +/−0.26. (beta1 = C/EBPbeta1, su-beta1 = sumoylated C/EBPbeta1).

After observing that phosphorylation of C/EBPbeta1 by Erk-2 enhances sumoylation *in vitro*, we mutated the Thr235 phosphoryation site to an alanine to determine if preventing phosphorylation of this residue would lead to a decrease in sumoylation. T7-C/EBPbeta1 or T7-C/EBPbeta1T235A with HA-SUMO-2/3 were transiently transfected into Cos-7 cells and immunoprecipitations were performed with T7 antibody beads. The middle immunoblot in Figure 3a is with the anti-HA tag antibody and demonstrates that mutant C/EBPbeta1T235A that cannot be phosphorylated at Thr235 is sumoylated to a lesser extent than C/EBPbeta1 (compare lanes 5 and 6). This is confirmed in lanes 8 and 9, which are the same samples only with the anti-C/EBPbeta antibody. The anti-phosphoThr235 C/EBPbeta antibody was used on the immunoblot on the left and lane three demonstrates that T7-C/EBPbeta1T235A does not react with the anti-phosphoT235 antibody because this residue has been mutated.

Figure 3b quantitates the difference in sumoylation between C/EBPbeta1 and C/EBPbeta1T235A. T7-C/EBPbeta1 and the T7-C/EBPbeta1T235A mutant were transiently transfected into Cos-7 cells along with HA-SUMO-2/3. The immunoblot with the anti-T7 tag antibody shown in Figure 3a demonstrates that the T7-C/EBPbeta1T235A mutant is sumoylated to a lesser extent than wild type T7-C/EBPbeta1 (compare lanes 3 and 2). Using the Odyssey system, we quantified the percentage of sumoylated T7-C/EBPbeta1 in the 75 kDa band compared to the non-sumoylated parent 52 kDa band and determined that there is 3.25 +/− 0.26-fold less sumoylated T7-C/EBPbetaT235A compared to wild type. This supports our findings in Figure 2 that phosphorylation of C/EBPbeta1 on Thr235 enhances sumoylation.

### Sumoylated C/EBPbeta1 does not induce senescence

**Figure 4:**
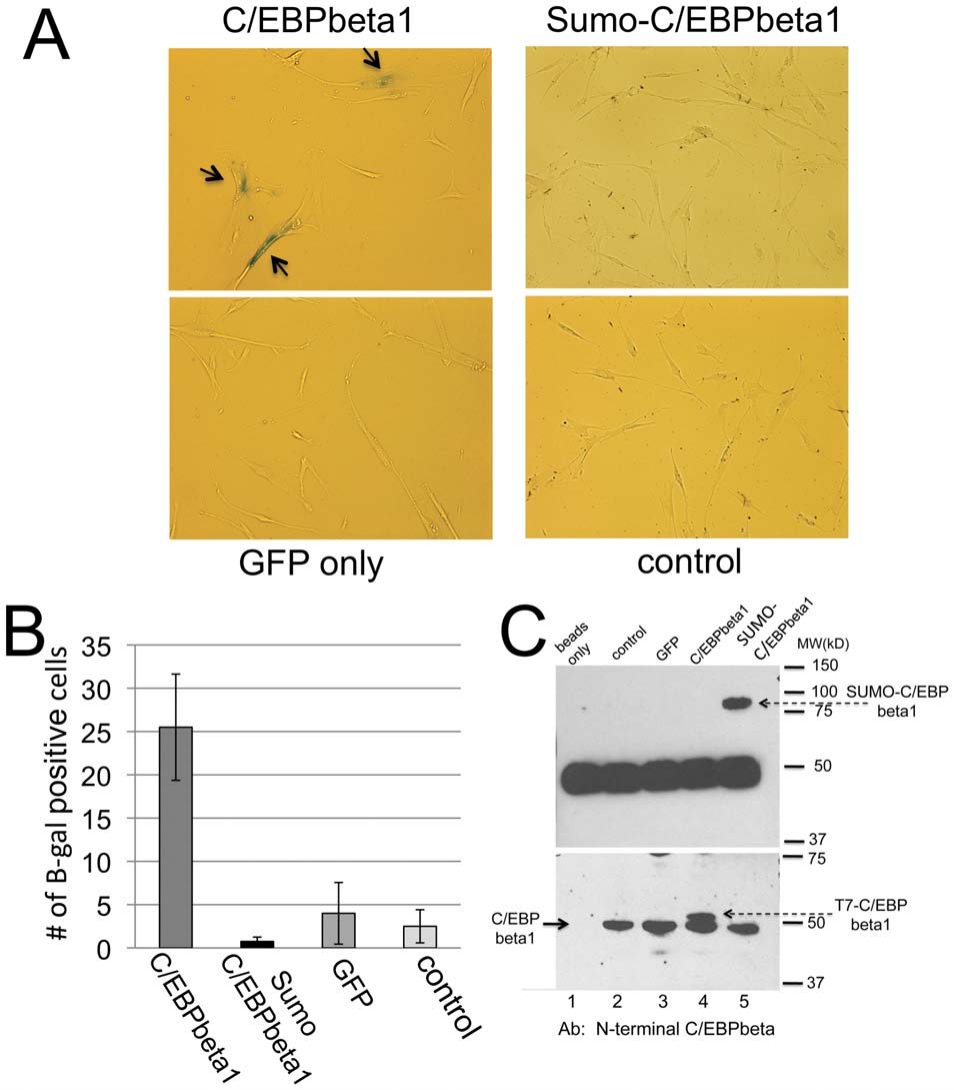
Sumoylated C/EBPbeta1 does not induce senescence. A. Equal cell numbers of the indicated cell lines were plated in 60 mm dishes and stained for senescence associated beta-galactosidase as per manufacturer's instructions (Cell Signaling Technology). Representative photomicrographs imaged with a light microscope are shown. B. Quantitative comparison of senescence associated beta-galactosidase positive cells. The experiment was repeated four times with equal cell numbers of the indicated cell lines ranging from 50,000–250,000 cells/60 mm dish. The density of plating did not affect the outcome. For each experiment the average number of beta-galactosidase positive (blue) cells in 10 fields was computed. Error bars indicated standard deviation of the mean. C. Whole cell extracts were prepared from the indicated WI-38hTert cells and analyzed by immunoblotting (bottom panel) or immunoprecipitated with T7 antibody beads (Novagen, upper panel) followed by immunoblotting. Immunoblot analysis was performed with N-terminal C/EBPbeta antibody developed in our lab and described in [7].

C/EBPbeta is required for oncogene-induced senescence, both by oncogenic Ras and activated Raf [20], [21]. We recently demonstrated that C/EBPbeta1 is the primary transactivator isoform responsible for the induction of senescence [14]. Sumoylated transcription factors frequently act as transcriptional repressors and we have previously shown that this is the case for C/EBPbeta1 [7]. Thus, we wanted to determine if sumoylated C/EBPbeta1 would be unable to induce senescence. To address this question we generated a chimeric protein in which SUMO2 is covalently linked to the C-terminus of C/EBPbeta1. We generated a retrovirus, LZRS-SUMO2-C/EBPbeta1/IRES/GFP, and infected WI38-hTERT cells (hTERT, the catalytic subunit of telomerase). WI38-hTert cells express endogenous C/EBPbeta1 and are able to undergo senescence. In fact as shown in Figure 4, exogenous expression of C/EBPbeta1 leads to a substantial increase in beta-galactosidase-positive, senescing cells. We quantitated the number of blue cells in 10 fields (at 10× magnification). A representative field is shown in Figure 4A and the average values from four independent experiments are shown in Figure 4B. There were on average 2.5 blue cells in the control population and a slight, insignificant increase to 4 blue cells in the GFP-only infected population. Infection with LZRS-C/EBPbeta1/IRES/GFP led to a substantial increase in beta-gal positive, blue cells. On average there were 25.5 blue cells/10 fields or a 10-fold increase in senescence. In contrast, infection with LZRS-SUMO2-C/EBPbeta1/IRES/GFP resulted in no increase in blue cells. Rather, the incidence of senescing cells dropped to less than one per 10 fields (average 0.75 cells/10 fields in four experiments). To confirm the expression of T7-C/EBPbeta1 and SUMO2-T7-C/EBPbeta1 we performed an immunoprecipitation using T7-tag antibody beads followed by western blotting with a C/EBPbeta1-specific antibody. As seen in the upper panel of Fig. 4C, lane 5, SUMO-C/EBPbeta1 migrates with an apparent MW between 75 and 100 kD, suggesting that the SUMO2 in the chimeric SUMO2-T7-C/EBPbeta1 protein is itself sumoylated. Because of the large amount of antibody heavy chain released from the T7-Ab beads, T7-C/EBPbeta1 could not be observed since it migrates at nearly the same apparent MW as the heavy chain. Thus, we detected T7-C/EBPbeta1 in the same whole cell extract used for the immunoprecipitation as shown in the lower panel of Fig. 4C, lane 4. Both T7-C/EBPbeta1 and SUMO2-C/EBPbeta1 were expressed at roughly equivalent levels. We conclude that sumoylation of C/EBPbeta1 effectively blocks its ability to induce senescence.

## Discussion

p52-C/EBPbeta1 is not observed via immunoblot of breast cancer cell lines with C/EBPbeta antibodies [17], Figure 1a, however breast cancer cell lines exhibit higher molecular weight bands that react with C/EBPbeta antibodies, including C/EBPbeta1-specific antibodies (Figure 1a). C/EBPbeta1 is known to be modified by post-translational modifications that can affect the apparent molecular weight of the protein via SDS-PAGE, including the post-translational modification sumoylation. C/EBPbeta1 is modified by SUMO-2/3 when transfected into Cos-7 cells and C/EBPbeta1 is the only isoform of C/EBPbeta known to be modified by sumoylation [7]. Modification by sumoylation adds 10–20 kDa to the apparent molecular weight of the target protein. Additionally, SUMO-2/3 itself contains a SUMO consensus site, so that SUMO-2/3 can be further sumoylated to form SUMO-2/3 chains. This can result in a ladder of higher molecular weight bands. We demonstrate that when T7-C/EBPbeta1 is expressed in breast cancer cells such as MDA231s, a ladder of higher molecular weight bands are observed with both a C/EBPbeta antibody and a SUMO-2/3 antibody, indicating sumoylation of C/EBPbeta1 (Figure 1b). Furthermore, we demonstrate that the higher molecular weight bands observed in the anti-C/EBPbeta1 immunoblot of breast cancer cells in Figure 1a are sumoylated C/EBPbeta1 (Figure 1c).

Phosphorylation oftentimes enhances sumoylation, and it is well known that signaling that activates phosphorylation of proteins is commonly activated in breast cancer cells. One example of this is the frequent activation of the Ras pathway in breast cancer cells. Ras pathway activation leads to the activation of numerous kinases known to phosphorylate C/EBPbeta on Threonine 235 (Thr235) including Erk-2 [34]), cdk2 [35], [36], and p38 [37], [38]. Therefore we wanted to determine whether phosphorylation of Thr235 in C/EBPbeta1 was enhancing sumoylation of C/EBPbeta1. Figure 2a demonstrates that full length C/EBPbeta1 is phosphorylated on Thr235 by Erk-2 *in vitro*, and Figure 2b reveals that this phosphorylation by Erk-2 on Thr235 enhances sumoylation of C/EBPbeta1, *in vitro*. Moreover, Figure 3b demonstrates that sumoylated C/EBPbeta1 in Cos-7 cells is phosphorylated on Thr235. To further examine the effect phosphorylation of C/EBPbeta1Thr235 had on sumoylation of C/EBPbeta1, we mutated C/EBPbeta1Thr235 to an alanine so that this residue could no longer be phosphorylated. Figure 3a and 3b demonstrate that this mutant is sumoylated to a lesser extent than wild type C/EBPbeta1, confirming that phosphorylation of Thr235 of C/EBPbeta1 enhances sumoylation of this protein.

Finally, we generated a constitutively sumoylated C/EBPbeta1 protein by fusing SUMO2 to the C-terminus of C/EBPbeta1. When introduced into human diploid fibroblasts WI38-hTERT, sumoylated C/EBPbeta1 failed to induce senescence whereas C/EBPbeta1 expression resulted in a 10-fold increase in senescing cells. Taken together, our results indicate that breast cancer cells may escape oncogenic Ras-induced senescence at least in part because Ras-Raf-ERK2 mediated phosphorylation of C/EBPbeta1 leads to its sumoylation which abrogates its ability to induce senescence.

We have previously shown that expression of oncogenic Ras expression can lead to the ubiquitination and degradation of C/EBPbeta1 in immortal MCF10A mammary epithelial cells [22]. It appears that there is more than one mechanism to prevent C/EBPbeta1 from inducing senescence, involving either sumoylation or ubiquitination. We know that C/EBPbeta1 is primarily sumoylated on K173 [7] but we do not know the identity of the lysines that are ubiquitinated prior to proteosome-mediated degradation. Thus, sumoylation and ubiquitination of C/EBPbeta1 may not be mutually exclusive, and both could be operative in transformed cells to escape oncogene-induced senescence.

## Materials and Methods

### Reagents

Unless otherwise indicated, reagents were purchased from Sigma Chemical Co. (St. Louis, MO, USA). The antibody directed against C/EBPbeta was obtained from Santa Cruz Biotechnology, Inc. (Santa Cruz, CA, USA). The anti-T7 tag monoclonal antibody was obtained from Novagen (Madison, WI, USA) and the anti-C/EBPbeta1-specific antibody raised to the 21 N-terminal amino acids present only in C/EBPbeta1 is Abcam 18F8. The C-terminal C/EBPbeta antibody used in Figure 1c is the Abcam 47A1 antibody. The anti-HA tag antibody, the SUMO-2/3 antibodies, the anti-rabbit and anti-mouse horseradish peroxidase (HRP)-conjugated secondary antibodies were obtained from Promega (Madison, WI, USA). The proteasome inhibitor MG132 (Calbiochem, San Diego, CA, USA) was resuspended in DMSO and used at a concentration of 50 uM. N-ethyl maleimide was resuspended in DMSO and used at a concentration of 5 mM. T7 tag antibody beads (Novagen).

### Cell lines

Tissue culture media was obtained from Life Technologies, Inc. (Carlsbad, CA, USA). Unless otherwise indicated, all tissue culture supplements were purchased from Sigma Chemical Co. (St. Louis, MO, USA). The MCF10A human mammary epithelial cell line was obtained from the American Type Culture Collection (ATCC). Cells were grown in a 1∶1 (v/v) mixture of Ham's F12 and Dulbecco's modified Eagle medium (DMEM) containing 2.5 mM L-glutamine and supplemented with 5% horse serum, 10 ug/mL insulin, 0.5 ug/mL hydrocortisone, 20 ng/mL epidermal growth factor, 100 ng/mL cholera toxin (Calbiochem Novabiochem, San Diego, CA, USA), 100 U/mL penicillin, and 100 ug/mL streptomycin (Life Technologies, Inc., Carlsbad, CA, USA). The human breast cancer cell lines MDA-MB-231, MDA-MB-468, HCC1954, SKBR3, BT474, MDA435, and T47D were obtained from the ATCC (Manassas, VA) and were maintained in Iscove's Modified Eagle media supplemented with 10% fetal bovine serum (FBS) from HyClone Laboratories (Logan, UT, USA), 10 µg/ml bovine insulin, 100 U/ml penicillin, and 100 µg/ml streptomycin (Life Technologies, Inc.). Cos-7 cells were a gift from Dr Steve Hann, Vanderbilt University and were maintained in DMEM plus 10% FBS (HyClone Laboratories, Logan, UT, USA). The phoenix-ampho packaging cell line was obtained from the ATCC with the permission of GP Nolan (Stanford University, Palo Alto, CA, USA) and has been previously described [39]. The packaging cells were maintained in DMEM supplemented with 10% heat-inactivated FBS (HyClone Laboratories, Inc., Logan, UT, USA), 1 mM sodium pyruvate, 2 mM L-glutamine, 100 U/mL penicillin, and 100 ug/mL streptomycin (Life Technologies, Inc., Carlsbad, CA, USA). WI-38 normal human diploid fibroblasts (a gift from the Dr. Hal Moses, Vanderbilt University) were maintained in Eagle's minimal essential medium (EMEM) containing 2.5 mM L-glutamine (Life Technologies, Inc., Carlsbad, CA), 10% fetal bovine serum (Hyclone Laboratories, Logan, UT), 100 U/mL penicillin, and 100 ug/mL streptomycin (Life Technologies, Inc., Carlsbad, CA). To generate WI-38-hTERT cells, pBABE-hygro-hTERT was purchased from Addgene (Cambridge, MA) and amphotropic retrovirus produced as described in [15]. WI38 cells were infected once and selected with 300 ug/ml hygromycin B. All cells were grown at 37 degrees Celsius in a humidified atmosphere containing 5% carbon dioxide.

### Cloning of recombinant constructs and virus preparation

T7-C/EBPbeta1-pcDNA3.1-His A was generated as described in [7]. C/EBPbeta1 is the only transactivator isoform produced from this construct due to mutation of the second in-frame ATG. Additionally, a perfect Kozak sequence was made centered around the first ATG. Generation of LZRS-T7-C/EBPbeta1-IRES-eGFP and T7-C/EBPbeta1T235A-pcDNA3.1-His A was as previously described [22]. Recombinant amphotropic retroviral stock generation and retroviral infection were performed as described in [15]. The hemagglutinin (HA)-tagged SUMO-2 expression vector was a kind gift of Dr. Ron Hay (University of St Andrews, St Andrews, UK). To create a C/EBPbeta1-SUMO2 fusion protein it was necessary to create a unique cloning site after the c-terminal amino acid (Cys) of C/EBPbeta before the stop codon. This had been previously achieved in our lab using a pRset-C/EBPbeta construct (pRset-LAP) [15] that was digested at a unique NruI site occurring 24 amino acids from the terminus of C/EBPbeta. A synthetic 82 bp oligonucleotide of the following sequence:

5′CGA GAG CTC AGC ACG CTG CGG ACC TTG TTC AAG CAG CTG CCC GAG CCG CTG CTG GCC TCG GCG GGT CAC TGC C**AG** **GCC** **T**TA G3′ was inserted at the NruI site to generate a unique StuI site (AGGCCT) immediately following the C-terminal Cys (TGC) codon. However, LAP begins at the second in frame ATG, and since a C/EBPbeta1 construct was desired, a Pst-HindIII C-terminal fragment of pRset-LAP(Stu) was cloned into pRset-C/EBPbeta1, replacing the cognate Pst-HindIII fragment. In order to perform the swap, it was necessary to eliminate a Pst site in the multiple cloning site of each of the pRset vectors. This was achieved by digesting either pRset-LAP(Stu) with XhoI and EcoRI or pRset-C/EBPbeta1 with Xho and Acc651, filling in the ends by treatment with Klenow, and reigating to drop out the Pst site.

At this point cloning was continued in BL21 cells that are dam/dcm methylase negative, because StuI is sensitive to methylation. pcDNA3-HA-SUMO2 was digested with EcoRI and BamHI and after filling in the ends with Klenow, the EcoRI-BamHI fragment encoding HA-SUMO2 was ligated to pRset-C/EBPbeta2(Stu) digested with StuI. The resultant pRset-C/EBPbeta1-SUMO2 construct was sequenced to verify the fusion protein sequence. Due to the cloning strategy used, 3 amino acids (QGS) are present between the C-terminal cysteine of C/EBPbeta1 and the N-terminal methionine of SUMO2 in the fusion protein.

The remaining cloning steps were performed in DH5alpha. pRset-C/EBPbeta1-SUMO2 was digested with EcoRI and partially digested with BamHI and the BamHI-EcoRI fragment encoding C/EBPbeta1-SUMO was transferred to pcDNA3.1hisC digested with BamHI and EcoRI. The resulting clone also contained 2 copies of a small BamHI-EcoRI fragment from the multiple clone site of pcDNA3.1 in a BamHI-EcoRI-BamHI orientation at the 5′ end of the BamHI-EcoRI fragment encoding C/EBPbeta1-SUMO2 which caused the C/EBPbeta1-SUMO2 fusion protein to be out of frame with respect to the T7-his epitope tag that was to be acquired upon transfer to pcDNA3.1. To correct this problem, an EcoRI fragment encoding C/EBPbeta1-SUMO2 was transferred to the EcoRI site of pcDNA3.1hisA. Finally, pcDNA3.1hisAC/EBPbeta1-SUMO2 was digested with HindIII, and after filling in the ends, digested with Not1 and transferred to the LZRS retroviral vector pBMN-GFP (Orbigen) that had been digested with EcoRI, and after filling in the ends, digested with Not1. The correct clone was verified by DNA sequencing.

### Transient transfections

Cos-7 cells were plated 18–24 hours prior to transfection so that the cells were 80–90% confluent at the time of transfection. Serum-free DMEM replaced complete media on cells 1 hour before transfection. 8 ug of total DNA was transfected into cells via 24 uL GenJet (SignaGen Laboratories, Gaithersburg, MD, USA) in serum-free media. After 5 hours, the media was changed to complete media. The cells were harvested two days post-transfection.

### Preparation of immunoprecipitations, cell lysates and immunoblot analysis

Confluent plates of cells were treated with 50 uM MG132 for 8 hours and 5 mM N-ethylmaleimide for 30 minutes for the immunoprecipitations. Immunoprecipitations were performed as described previously (Eaton and Sealy, 2003) with the following exceptions: the immunoprecipitations were for 15 minutes and 50 uM MG132 and 5 mM N-ethylmaleimide were included in the immunoprecipitation buffer. Cell lysates were prepared from 100 mm dishes of 90% confluent cells as described previously [7]. Relative protein concentrations were determined using the Protein Assay Reagent (BioRad Laboratories, Hercules, CA, USA) as per the manufacturer's instructions. Equal amounts of protein were loaded onto 10% SDS-PAGE and separated by electrophoresis. The proteins were transferred to an Immobilon P or Immobilon FL filter and the blots were processed as described previously [17]. After the nonspecific binding sites were blocked, the blots were inclubated with primary antibody (C-terminal C/EBPbeta at a 1∶5 000 dilution; T7 at 1∶10 000, N-terminal C/EBPbeta at 1∶2 000) in TBS-T (100 mM Tris pH 7.5, 150 mM NaCl, and 0.05% Tween-20) containing 0.5% nonfat dried milk (NFDM) for 1 hour at room temperature. The blots were washed with three successive changes of TBS-T containing 0.5% NFDM at room temperature for 20 minutes and incubated with a HRP-conjugated goat anti-rabbit (1∶5 000 or 1∶2 000 dilution) or a HRP-conjugated goat anti-mouse antibody (1∶10 000 dilution) as described above for an additional hour. The blots were then washed with three successive changes of TBS-T solution for 15 minutes and the signal was detected by chemiluminescence using SuperSignal West Pico reagent (Pierce, Rockford, IL, USA) and autoradiography with Kodak X-OMAT film (Rochester, NY, USA). Alternatively, the LI-COR ODYSSEY infrared imaging system (Lincoln, Nebraska) was used for immunoblot analysis. Quantitation was performed as per manufacturer's instruction.

### Cross-linking of C/EBPbeta1-specific antibody to protein A beads

The serum from the rabbit polyclonal C/EBPbeta1-specific antibody raised to a 16 amino acid peptide corresponding to the first 16 amino acids in human C/EBPbeta1 described in [17], was used in the cross-linking. We began by ammonium sulfate precipitating the antibody out of the serum. Serum containing approximately 4 mg antibody was first clarified. An equal volume of saturated ammonium sulfate pH 7.5–8.0 was slowly added dropwise into the clarified serum at 4 degrees Celsius. The serum/ammonium sulfate solution was mixed frequently during the addition of ammonium sulfate. The serum/ammonium sulfate solution was rotated at 4 degrees Celsius overnight. The next day the serum/ammonium sulfate solution was spun in an HB-4 swinging bucket rotor at 3000×g for 30 minutes at 4 degrees Celsius. The supernatant was removed and the pellet containing the antibody was resuspended in 1× PBS. The PBS/antibody solution was then transferred to dialysis tubing and dialyzed in 1× PBS overnight at 4 degrees Celsius. The solution in the dialysis tubing was clarified the following day. To cross-link the antibody to the protein A agarose, 2 mL of protein A agarose slurry (Invitrogen) was washed in 1× PBS and collected. 4 mg of the ammonium precipitated antibody was mixed with the beads for 1 hour at room temperature. The beads were then washed with sodium borate and the antibody was cross-linked to the beads with 20 uM dimethylpimelimidate in sodium borate rocking for 30 minutes at room temperature. The cross-linking was quenched by rocking the beads in 0.2 M ethanolamine pH 8.0 for 2 hours at room temperature. Finally, the beads were washed in 1× PBS and stored at 4 degrees Celsius.

### In vitro phosphorylation and sumoylation

5 ug of purified rat C/EBPbeta1 (Lap1) protein was incubated with 100 uM ATP, 10 mM magnesium chloride, and protease and phosphotase inhibitors (10 uM sodium vanadate,10 mM sodium molybdate, 10 mM beta-glycerophosphate, 1 ug/mL aprotinin, 1 ug/mL leupeptin, 1 ug/mL pepstatin, and 1 mM phenylmethylsulfonyl fluoride). One half of this original sample was incubated with 0.5 ug active, purified Erk-2 (Upstate/Millipore) for 1 hour at 30 degrees Celsius. The other half of the sample was not incubated with Erk-2. Half of the sample incubated with Erk-2 (so one quarter of the original) and half of the sample not incubated with Erk-2 were then incubated with the sumoylation machinery for 1 hour at 37 degrees Celsius. The purified sumoylation machinery included 0.05 ug/uL human E2 conjugating enzyme (Ubc9), 0.05 ug/uL human SUMO-3 peptide, and 0.0075 ug/uL E1 activating enzyme (SAE I/II) (LAE Biotechnology Co.). Sumoylation was performed in the presence of 0.1 mM ATP, the protease and phoshotase inhibitors (10 uM sodium vanadate,10 mM sodium molybdate, 10 mM beta-glycerophosphate, 1 ug/mL aprotinin, 1 ug/mL leupeptin, 1 ug/mL pepstatin, and 1 mM phenylmethylsulfonyl fluoride), and 1× SUMO Buffer (LAE Biotechnology Co.). After sumoylation, 2× SDS sample buffer was added to the four different samples. The samples were boiled for 5 minutes and stored at −70 degrees Celsius until subjected to SDS-PAGE.

### Senescence associated beta-galactosidase assay

50% confluent WI-38 cells in 60 mm plates were fixed and stained with the senescence beta-galactosidase staining kit per the manufacturers instructions (Cell Signaling Technology, Beverly, MA).
